# Neonatal Surgical Morbidity and Mortality at a Single Tertiary Center in a Low- and Middle-Income Country: A Retrospective Study of Clinical Outcomes

**DOI:** 10.3389/fsurg.2022.817528

**Published:** 2022-02-03

**Authors:** Md Samiul Hasan, Nazmul Islam, Ashrarur Rahman Mitul

**Affiliations:** Pediatric Surgery Division, Bangladesh Shishu Hospital and Institute, Dhaka, Bangladesh

**Keywords:** pediatric surgery, neonatal surgery, challenges, LMIC, expectations

## Abstract

**Background:**

The most challenging and demanding issue in Pediatrics and Pediatric Surgery is to deal with neonatal surgery which almost always involves emergency neonatal surgical conditions. Emergency neonatal surgery most often involves congenital anomalies. More than 90% of these anomalies occur in low- and middle-income countries (LMICs) like Bangladesh. This considerable load of patients and inadequate resources in their management continue to be an unconquerable challenge for pediatric and neonatal surgeons in this region. We aim to review the challenges and constraints influencing the outcomes of emergency neonatal surgery which will guide to propose expectations from the global community.

**Method:**

We reviewed hospital records of neonates admitted to a tertiary care pediatric hospital between January 2016 and December 2020. Demographic and clinical data were extracted using a questionnaire and analyzed using SPSS 25.

**Results:**

A total of 3,447 neonates were admitted during the five-year study period. More than 80% of the patients had at least one prenatal ultrasonography (USG) scan, but less than 10% had a prenatal diagnosis. More than 70% of the anomalies of the patient involved the gastrointestinal tract and abdominal wall. Overall mortality was an alarming 14.6%. Gastroschisis (>90%) and esophageal atresia (>85%) mainly contributed to this high mortality. The challenges detected in this review were the absence of a prenatal diagnosis, limited access to intensive care facilities, unavailability of parenteral nutrition, inadequate monitoring, and hospital-acquired sepsis.

**Conclusion:**

Emergency neonatal surgery contributes to a significant proportion of neonatal mortality. A holistic approach is essential to improve the situation, including the infrastructure and human resource development, identification of causes, and implementation of preventive measures to reduce the patient load. Global collaboration remains to be a vital factor to mitigate these multifactorial constraints.

## Introduction

Neonatal surgery has remarkably evolved in developed countries over the past decades, significantly reducing morbidity and mortality. This evolution is mainly due to improved perioperative care, which is sophisticated but expensive, not affordable by low-income countries (1–3). Unfortunately, more than 90% of the neonatal surgical patient load is in the low-and middle-income countries (LMICs) (4, 5). When poverty is one of the dominant factors contributing to this enormous burden, the treatment of these children makes the situation worse. This vicious cycle of poverty and insufficient health care facilities for surgical neonates results in a substantial difference in the outcome of neonatal surgery between high-income and low-middle-income countries (1–3, 6–8). The data available are only based on inpatient admitted babies. However, a large proportion of the neonates in LMICs have no access to surgical care, which further magnifies the outcome differences (5, 9).

The actual data regarding neonatal surgical patient load in Bangladesh is not available, but this burden is increasing and contributing significantly to the causal list of neonatal mortality. Optimum surgical care could avoid at least two-thirds of this mortality (5, 7–10). United Nations sustainable development goal (SDG-3) targets to end preventable neonatal death by the year 2030 (11). Indeed, this is not achievable without improving surgical care for neonates in resource-limited places. We aim to review the challenges and constraints influencing the unusually higher mortality of neonatal surgery, which will guide us to propose expectations from the global community.

## Materials and Methods

### Study Design

This was a retrospective descriptive study. We reviewed the death registry of the pediatric surgery division from January 2016 to December 2020. Babies with age ≤ 28 days at admission were included in this study. We have looked into the Hospital records of the babies identified from the death registry. We have also described the hospital settings in which surgical neonates get operative and perioperative management.

### Study Setting

This study was conducted in the division of pediatric surgery of Bangladesh Shishu Hospital and Institute, the largest children's hospital of the country dedicated solely to pediatric patients. With more than 650 beds, this hospital is the largest referral center for children from all over the country.

The division of pediatric surgery has five units with 105 dedicated beds in two wards, but surgical patients can also get admitted to medical wards and cabins. Out of the 105 beds, 45 beds are nonpaying, while other beds cost 700 BDT (±10USD) per day. The cabins cost five times more. Parents also have to pay for the investigations and medicines separately. We have 15 faculties specialized in pediatric surgery at different levels of their practice. Assistant professors have a minimum of 1 year of clinical experience after completing their post-graduation. Associate professors and professors have 5 years and 10 years of clinical experience, respectively, after post-graduation. This hospital runs a 5-year long structured Master of Surgery (MS) residency course and currently has 30 post-graduate trainees at different levels of their training. The pediatric anesthesiology department consists of six faculties and eight medical officers. The operation theater complex has four theaters equipped with a general anesthesia setting and one theater for short procedures. The hospital has a 14-bed pediatric intensive care unit (ICU) and a 16-bed neonatal ICU.

All neonates with a surgical condition get admitted into the wards or cabins according to availability and affordability. The distance between the beds in the wards is less than two feet, and there is no temperature regulating system available in the wards. The monitoring of the neonates before and after the operation is manual, and there is no digital patient monitoring device available in the wards and cabins. Routine surgeries are performed during the regular office hours (8.00 a.m. to 2.30 p.m.), mainly by the consultants or senior residents under supervision. Emergency operations are usually performed late in the evening by the residents in the last year of their post-graduate training, mostly without direct supervision. Nursing facilities are grossly inadequate, and the nurse-patient ratio is never more than 1:15 during any 24-h period.

### Data Collection

We have pre-designed a data collection form to extract the data from the hospital records. The form was specially designed to identify the limitations in providing optimum surgical care to neonates, which included demographic and clinical variables and facilities available for the treatment. The total admission, surgeries performed, and mortality rate data were collected from the monthly audits of the surgery division.

### Data Analysis

From the data collection form, data were entered into SPSS version 22 for analysis. Categorical data were presented as frequencies, and numerical data were presented as mean ± standard deviation.

### Ethical Clearance

Ethical clearance for this study was taken from the ethical review committee of Bangladesh Shishu Hospital and Institute.

## Result

Out of 3,447 neonates, 503 died during the study period, leading to a mortality rate of 14.6%. More than 54% of these neonates were premature. The minimum gestation in this study was 27 weeks, and the mean gestational age was 35.38 ± 2.34 weeks. The mean birth weight was 2.32 ± 0.44 kg (range 1.20 to 3.50 kg). Male babies were more (64.4%) than females. Anorectal Malformations and Gastroschisis were the commonest of the anomalies (Table 1). Only two babies had a prenatal diagnosis, though more than 85% of mothers had at least one ultrasound scan during pregnancy. Both of them had bilateral hydronephrosis due to the posterior urethral valve.

**Table 1:** Common diagnoses of babies who expired.

**Diagnosis**	**Frequency**	**Percent**
ARM	69	13.7%
Rectourinary fistula	64	
Rectovestibular fistula	3	
Perineal fistula	2	
Gastroschisis	67	13.3%
Simple	65	
Complicated	2	
Hirschsprungs Disease	55	10.9%
Classic rectosigmoid	39	
Long segment	1	
Level not identified	15	
Jejunoileal atresia	52	10.3%
Jejunal	38	
Ileal	14	
Meconium ileus	42	8.3%
Uncomplicated	27	
Complicated	15	
Spontaneous Intestinal perforation	38	7.5%
Omphalocele	26 24	5.2%
Major		
Minor	2	
Esophageal atresia (with lower pouch fistula)	24	4.8%
Duodenal atresia	23	4.6%
Congenital diaphragmatic hernia (left-sided)	15	3%
Necrotising enterocolitis	13	2.6%

The average duration from hospital admission to definite management was 26.4 h. Only 34 (6.8%) babies managed to access the neonatal intensive care unit (NICU). No one received total parenteral nutrition (TPN).

Emergency operations were performed in 340 (67.6%) babies. One hundred three (20.5%) babies died before operation due to severe hypovolemia and sepsis. Only 60 babies died after a routine operation. Out of the 503 babies, 445 (88.5%) babies had congenital malformations. The most common cause of death was sepsis (Table 2). Gastroschisis and esophageal atresia were associated with the highest mortality (Table 3).

**Table 2:** Common causes of death.

**Cause of death**	**Frequency**		**Percent**
Sepsis: Preoperative	103	Total 405	80.5%
Postoperative	302		
Cardiogenic shock	43		8.6%
Respiratory failure	38		7.6%

**Table 3:** Diagnoses with the highest mortality.

**Diagnosis**	**Mortality**
Gastroschisis	93.06% (67/72)
Esophageal atresia	88.89% (24/27)
Jejunoileal atresia	21.05% (52/247)
ARM	17.56% (69/393)
Intestinal perforation	16.74% (38/227)

## Discussion

Bangladesh is having one of the highest non-natal mortality rates globally. More than 62,000 neonates die here every year (11). The contribution of neonatal surgery in this mortality is unknown, but clearly, the burden is increasing (7, 8). Despite this rise, neonatal surgery fails to receive enough attention and importance in health care policies and surgical neonates remain the most neglected cohort during policy-making. Under-recognition is responsible for this high mortality. Several preventable causes are responsible for this gloomy scenario and ultimately the surgical neonates are the victims.

The mortality rate in this study is close to the reports published from two other high-volume centers of the country, which reflects the similarity in the neonatal surgical setups (7, 8). Though data are not available, we can assume that the situation is worse in smaller centers. The scenario is similar in other LMICs and reflects an unacceptable difference in neonatal surgical mortality with high-income countries (HICs) (12). This difference is not due singularly to the surgery itself; unavailability of expensive perioperative management also has a major contribution to it. (12, 13). The expensive perioperative setup and inadequate data supporting its necessity may be the causes of the negligence about neonatal surgery in policymaking in LMICs.

More than half of the expired neonates were premature, and the mean birth weight was below average. Prematurity and low birth weight are two independent variables for neonatal surgical mortality. A report by Puri et al. showed that prematurity and low birth weight increase the odds of neonatal surgical mortality by 3.38 and 3.41 times, respectively. However, with the advanced perioperative support system, these are no longer the predictors of mortality in HICs (14).

Antenatal diagnosis of complex congenital anomalies allows planning for delivery and early post-natal treatment, associated with a better outcome. However, developing countries still lag in this field due to the unavailability of instruments and expert personnel (12). Our study found this statement to be real; only two babies had a prenatal diagnosis, though more than 85% of the mothers had at least one ultrasound scan during pregnancy. It resulted in the delay in diagnosis, referral, and treatment of the babies with complex congenital anomalies, and more than 20% of the babies died before operation due to unsuccessful resuscitation.

Neonatal surgery is very delicate and sophisticated in every aspect. Prolonged duration of surgery, per-operative blood loss, and hypothermia after surgery adversely affect the outcome. In our study, 67.6% of babies died after emergency surgery, primarily performed late in the evening by residents without supervision. A massive load of scheduled surgery and limited theater and personnel availability do not allow rescheduling of emergency surgery during office hours.

Only 3.8% of babies had access to NICU. All of them had esophageal atresia and congenital diaphragmatic hernia. They had to wait for at least 3 days in the ward to get this access, which delayed their surgical treatment and contributed to the poor outcome. Even in the NICU, no baby got TPN, though it was necessary for most of them. TPN is not available here. Moreover, central venous access requires extra time, is expensive, and is challenging to manage in the wards where 4–5 nurses manage more than 50 babies. This affected the outcome of babies with gastrointestinal surgery, especially babies with gastroschisis.

The most common diagnoses of the expired babies were Anorectal Malformation (ARM) and Gastroschisis; this might be due to the higher incidence of these anomalies in this region. Reports from other institutes of Bangladesh and India reflect this fact (7, 8, 15). The highest percentage of babies died with Gastroschisis and Esophageal atresia. The leading causes are delays in the definite treatment and unavailability of the intensive care facilities. As our hospital does not have a maternal wing, all the babies are outbound. Moreover, the absence of prenatal diagnosis causes the delay in the treatment. A report from a corporate hospital in Bangladesh showed that the timely provision of sophisticated perioperative care could improve the outcome of these babies to an acceptable level (16).

Sepsis was the most common cause of death in this study. It is actually the final result of limitations in diagnosing and managing the surgical neonates in LMICs as described by Mitul AR (13). It is often frustrating and tiring for the surgeons working in these regions when all the efforts go in vain.

There was no difference in outcome between the babies admitted in paying and non-paying beds. The treatment protocol is the same in both cases. Whenever the family fails to afford the treatment cost, the attending surgeon organizes the beds.

As the study was conducted at the highest referral center for neonatal surgery, which deals with the most significant fraction of the neonatal surgery of the country, the results could be generalized to the whole country. Therefore, planning and implementing a development program in this center would certainly improve mortality as a whole. The problems we have identified in this study could be broadly categorized into two groups: (A) Insufficient infrastructure and (B) Human resource crisis. We have also outlined a possible solution requiring multisectoral collaboration involving hospital authority, government, and non-government organizations (NGOs).

It is not necessary that we need all sophisticated modern technologies here, which would be unreasonable. Instead, locally customized, affordable technologies are. For example, if we could provide a separate ward for surgical neonates with facilities for temperature and infection control, we could reduce the incidence of sepsis and related mortality. An addition of patient monitoring systems here would allow early detection and treatment of critical neonates. Here, the hospital needs funds from the government or NGOs.

The ratio of healthcare providers (including surgeons, anesthetists, nurses, and auxiliary staff) to patients is significantly below the minimum threshold level. This area needs urgent consideration from the hospital authority and the government. Maintaining this ratio at an acceptable level would allow roster allocation of the consultants during emergency hours and optimum monitoring and treatment of the patients.

Multicenter collaboration with advanced centers worldwide should be considered for exchanging knowledge and skill. To limit the patient load, research should also be encouraged to identify the risk factors for the high incidence of congenital anomalies in the country.

All maternity centers should be integrated or affiliated with a neonatal care unit for early identification and to prevent the dillydallying with these delicate beings. Trained personnel should also be available to be able to handle these babies while transporting to a higher center.

For an LMIC, it is truly impossible to overcome these challenges individually without cooperation from international bodies like the WHO, UNICEF, and other aid agencies. Regional and international collaboration need to be an integral part. Recognition of all these issues is of paramount importance while addressing this field of medicine. It is also of utmost importance that a central controlling body under the supervision of the government itself is to be formed without depending on the commercial bodies and institutions. Bangladesh government has launched National Newborn Health Programme to end preventable child death by 2035. With the same purpose, UNICEF is providing “Newborn bundle” care (11). Unfortunately, neonatal surgery got no attention in any of these, though it is impossible to achieve the goal without proper attention to neonatal surgery.

## Conclusion

Surgical neonates significantly contribute to the total neonatal mortality of developing countries like Bangladesh, a considerable proportion of which is preventable by appropriate perioperative care. Still, neonatal surgery does not get its due attention it the national health policy. If it continues to go like this, the achievement of SDG 5 will not be possible.

## Data Availability Statement

The original contributions presented in the study are included in the article/Supplementary Material, further inquiries can be directed to the corresponding author/s.

## Ethics Statement

The studies involving human participants were reviewed and approved by Institutional Review Board, Bangladesh Shishu Hospital and Institute. Written informed consent to participate in this study was provided by the participants' legal guardian/next of kin.

## Author Contributions

MH and NI was involved in data collection and manuscript preparation. AM was involved in overall supervision and final editing of the manuscript. All authors contributed to the article and approved the submitted version.

## Conflict of Interest

The authors declare that the research was conducted in the absence of any commercial or financial relationships that could be construed as a potential conflict of interest.

## Publisher's Note

All claims expressed in this article are solely those of the authors and do not necessarily represent those of their affiliated organizations, or those of the publisher, the editors and the reviewers. Any product that may be evaluated in this article, or claim that may be made by its manufacturer, is not guaranteed or endorsed by the publisher.
